# Patients' and Health Professionals' Experiences of Group Training to Increase Intensity of Training after Acquired Brain Injury: A Focus Group Study

**DOI:** 10.1155/2021/8838038

**Published:** 2021-01-07

**Authors:** Gunhild Mo Hansen, Iris Brunner, Hanne Pallesen

**Affiliations:** Hammel Neurorehabilitation Centre and University Clinic, Aarhus University, Aarhus, Denmark

## Abstract

**Background:**

Increased intensity of training in the subacute phase after acquired brain injury facilitates plasticity and enhances better function. Group training can be a motivating factor and an effective means of increasing intensity. Reports on patients' and health care professionals' experiences on increasing the amount of active practice through group training during in-patient rehabilitation after acquired brain injury have been limited.

**Methods:**

Two focus groups, patients and health care professionals, participated each in two interviews, before and after implementation of the Activity block, i.e., 2-hour daily intensive group training. The data from the interviews were analyzed from a phenomenological perspective.

**Results:**

Three categories emerged from the data analyzes (i) training intensity, (ii) motivation and meaningfulness, and (iii) expectations and concerns. Both groups experienced that the training after implementation of the Activity block had become more intense and that motivation was increased induced by the group setting. Also, both groups found self-management enhanced. Some challenges were also reported. Patients expressed concerns to finding a balance between rest and activity, while the health professionals mentioned practical challenges, i.e., planning the content of the day and finding their role in the Activity block.

**Conclusion:**

Activity block benefitted a heterogeneous group of patients with acquired brain injury and was perceived as an overall positive experience by patients and health personnel. Matching the training to the individuals' need for support, finding a balance between rest and activity and using tasks that support patients' motivation, appeared important.

## 1. Background

After a stroke or other acquired brain injury (ABI), many patients are admitted to in-patient rehabilitation in order to improve their function. Rehabilitation usually takes place during the first weeks after stroke when most changes due to plasticity can be expected [1]. To facilitate plasticity, it is critical to provide intensive, task-oriented practice. In studies with humans and nonhumans, it has been demonstrated that increased intensity of training results in better function [2–5].

In rehabilitation practice, however, this seems difficult to achieve. Repeatedly, low activity levels have been found during in-patient rehabilitation. According to Bernhardt et al. [6], patients were only active for 13% of their day, excluding night time. These findings were confirmed in more recent studies from other rehabilitation wards, where patients were observed to be sedentary 74% of the day [7] and engaged in nontherapeutic activities 78% of the day [8]. There seems to be a large potential to increase intensity, but limited resources counteract this potential. An increasing number of stroke patients are to be expected due to the aging population, while on the other hand, a shortage of qualified health professionals has been predicted by the WHO [9]. There is an increasing need for effective treatment strategies and an effective treatment organization.

Group class or group training could be a viable and effective means of increasing intensity during in-patient rehabilitation. Group therapy during in-patient rehabilitation is safe and equally effective in patients with moderate to severe gait impairment [10]. Groups of circuit classes for gait training were found to be safe to conduct and improved gait speed more than individual physiotherapy [11]. Patients were also found to spend more time in active task practice in circuit classes compared to individual training [12]. However, increased intensity is not always translated into better function. English et al. [13] compared two different models of increased therapy time, circuit class training provided 5 days a week and individual physiotherapy provided 7 days a week, to standard care. In this large RCT including 283 patients, no difference in walking distance and only a nonsignificant difference in length of stay was found. Nevertheless, recent reviews by English et al. [13] and Bonini-Rocha [14] suggest that circuit class training can be beneficial in terms of gait and mobility after stroke.

Group training is not only a means of increasing the amount of active practice. It can also offer beneficial effects on psychosocial well-being. In earlier research, patients highly valued the encouragement and the mutual support that group training can offer [15]. Observing others in the same situation was experienced as motivating. However, the need for individual adaptation was emphasized. Therapists regarded circuit class training as a valuable add-on to individual therapy and acknowledge the psychosocial benefits for their patients [16]. Yet, there were ambivalent views regarding patients with more severe impairments and concerns that the possibility for individual treatment in terms of quality of movement may be reduced. Implementation of Activity block, i.e., group training for two hours five days a week, was to be started up in a subacute neurorehabilitation ward for patients with complex impairments after acquired brain injury. Our aim was to explore how both patients and health professionals experienced (pros and cons) the change from individual treatment to group treatment.

## 2. Methods

### 2.1. Study Design

A qualitative approach was chosen which facilitates the researcher to enter the world of the participants and gain insight into their thoughts and feelings [17]. The qualitative interview is a uniquely sensitive and powerful method for capturing the experiences and lived meaning of the participants' everyday world. The interviews allow the participants to convey to others their situation from their own perspective and in their own words [18]. The interviews were inspired by the phenomenological theoretical perspective and aimed to explore the participants' perceptions, interpretations of their own experiences, and everyday reasoning.

Stroke individuals were regarded as experts for their own experiences.

### 2.2. Setting

A specific ward at a hospital offering specialized neurorehabilitation for patients in the early subacute phase framed the setting. The hospital has several sensory gardens, nearby forests, and well-equipped therapy rooms that make varied group training possible. The patients at the specific ward were in the early subacute to late subacute phase, diagnosed with stroke, traumatic brain injury, or other acquired brain injuries.

### 2.3. Traditional Training

Traditional training consists of a multidisciplinary determined effort to support self-management in daily life activities and one to one or two to one therapy in different therapy settings in order to facilitate better performance in specific functions that can support this.

Patients participate in group training with specific focus, i.e., task-oriented-training, when estimated as relevant and possible to complete with little support.

### 2.4. Central Concepts of the Activity Block

An overall focus of the new initiative was to offer all patients in the ward participation in a two-hour group activity training, called the Activity block, at a set time every morning. An important goal was to adjust continually the training program in order to pursue the individuals' rehabilitation goals. The group training was to be carried out by physiotherapists and occupational therapists, supported by nurses, and take place in the hospital's joint training area. Each weekday had a special focus, see Table 1, and training consisted of various approaches addressing all levels of the ICF [19], e.g., sit to stand from various heights, activities aiming to better strength and aerobic capacity, brain education classes, kitchen activities, and outdoor activities.

### 2.5. Selection of Participants

The patient participant group consisted of seven individuals, aged 26-71 suffering from a confirmed diagnosis of brain injury with moderate or severe disability. The inclusion criterion is being able to join the Activity block. The exclusion criteria are cognitive and communication disability making it impossible to share experiences. All patients from a specific ward, seven patients at the time of the first interview and six patients at the second interview, were invited to participate in focus group interviews. Administrative staff at the ward selected and verbally informed patients who matched the inclusion criteria. All seven patients provided informed consent to participate in the study. The patients were interviewed for the first time one week before implementation of the Activity block, while still participating in standard rehabilitation. Patients were interviewed a second time after having participated in the Activity block for approximately two weeks (May and June 2019). In order to elucidate the research question, we selected a strategic sample of health professionals representing the close team (nurses, occupational therapists, and physiotherapists) with various experience within the field of neurorehabilitation. Health professionals participated in the first interview two weeks before the Activity block started (May 2019). The second interview was conducted three and a half months later (September 2019) to allow for a nuanced experience over an extended period of time.

### 2.6. The Focus Group Interviews and Interview Guide

Rehabilitation practice before and after the implementation of the Activity block was explored by semistructured focus group interviews. The rehabilitation professionals' perspective allowed for exchange and elaboration of experiences and ideas among colleagues [20]. Patients were encouraged to express their experiences with different modes of training and discuss them with fellow patients. The interview guide was jointly developed by all authors with main topics on (i) the participants' and (ii) the professionals' experiences, reflections, and performance, see interview guide Table 2. These topics were discussed before implementation of the Activity block and had slight variations after the implementation of the Activity block. Four focus group interviews were carried out by the authors. The interviews were audio-recorded and transcribed verbatim.

### 2.7. Procedures

The focus group interviews with the patients were conducted on the ward in a separate meeting room, all participants sitting around an oval table. The focus group interviews with health professionals were carried out in the same setting. The interviews were led by a moderator, the last author (HP), while the first (GMH) and second (IB) authors of this article asked supplementary questions.

Before the interview was initiated, the moderator clarified the process. The moderator followed a standardized procedure of organizing, conducting, recording, and handling the interview.

The interviews with patients took place ahead of the focus group interviews of the health professionals. Concerns and expectations brought forward by the patients could thereby be elaborated and discussed in the subsequent interviews with therapists. The data from the two different groups of interviews were analyzed separately.

### 2.8. Data Analysis

A five-step analytical approach with inspiration from the phenomenological approach of Giorgi [21] and Kvale and Brinkmann [22] framed the analysis. In Step 1, reading and rereading the whole interview in order to gain a sense of the whole. Both the first and last author read the interviews in printed forms and relistened to the audio recordings to ensure accuracy in the transcriptions. In Step 2, natural “meaning units,” as they were expressed by the interviewees, were identified. The interviews were separately reread and categorized into meaning units by the two first and the last authors together. Meaning units were formed each time the authors reached consensus. In Step 3, the dominating themes in the meanings units were identified. The researcher endeavored to thematize from the interviewed person's point of view, as the researcher understood it. The identified meaning units were transformed into temporary categories and patterns that represented the informants' natural attitude expressions, as understood by the authors. This was done by manually tracing, identifying, and reaching consensus about provisional categories and patterns in the empirical data. In Step 4, the meaning units were questioned based on the research questions. Next, the meaning units were reread, and the informants' natural attitude expressions were transformed into socially and psychologically sensitive expressions. After reaching consensus about the meaning of the quotations, we then grouped the meaning units into categories that represented the essences of the phenomenon, moving from situated structures to a general structure. The meaning units were moved around until all three authors were agreed that they were all in the right categories. The most representative and clarified meaning units were then organized. In Step 5, the nonredundant themes were condensed into descriptive statements. In this final step, the meaning units were synthesized and described in a final set of themes.

First, themes were identified in structures as they spontaneously appeared in the data. The first and last authors concentrated all the themes together. Thereafter, the themes were shared between all the authors. During this iterative writing process, all three authors continually evaluated and discussed the variations in the themes to ensure credibility, transparency, and trustworthiness of the emerging results.

## 3. Ethical Considerations

The study was completed in accordance with the Helsinki Declaration of 2013 [23]. The Regional Ethics Committee for Central Jutland approved the study, registration number 1-16-02-840-17. Informed written and verbal consent to participate and access to medical records were obtained from the patients. Participation was voluntary, and withdrawal was possible at any time without changes to ongoing or future rights to treatment. To preserve anonymity, identifiable places or situations related to the participants have been changed.

## 4. Findings

A strategic sample of 13 individuals, seven patients and six health professionals, were included. Patients were between 26 and 71 years; health professionals were between 26 and 58 years. See Tables 3 and 4 for further details. All were well acquainted with the group training.

A total of four focus group interviews were conducted, two with patients and two with health professionals. Each interview lasted between 27 and 49 minutes. Three main themes emerged based on the quotations and processing of the data: theme 1, “training intensity”; theme 2, “motivation and meaningfulness”; and theme 3, “Expectations and concerns”. Text condensed from the interviews was translated from Danish to English. These three themes reflect experiences from patients and health professionals, prior to and after initiating the Activity block. The findings are presented as categories condensed from the interviews, see Table 5, and are further supported by patients' and health professionals' quotations involving most of the participants and are presented with informant information (patient or health professional and their number).

### 4.1. Training Intensity

#### 4.1.1. Before Implementing the Activity Block

One focus both among patients and professionals was on the amount of training that was offered. Patients expressed that there was time for more training in their day:

Patient 2: “I think that I get too little training here…I am sitting passively most of the time and just stare…”.

There was also a consideration about the need for rest, and the patients emphasized the importance of finding the right balance between rest and activity:

Patient 6: “It is of outmost importance for my training, that I will not get tired, because then I will train wrongly”.

Patient 3: “I would like to have much more training, not just half an hour, barely, before and after midday…I feel that I am almost starting from scratch each time, but I don't, and I know that because I have come a long way. Therefore it is working what you do to me, but I feel that it is going too slowly”.

The health professionals focused on amount of training and expressed their thoughts about the concept of intensity and the content of training in the following way:

Professional 5: “Intensity is of course both having a lot to do during the day, but intensity is also the content in the one session where you are together with the patient”.

Professional 4: “There are more layers, we want both increased intensity over the day, and we also want more intensity in each therapy session”.

One nurse described intensity as part of recurring activities during the day and emphasized a need of finding a balance between organizational constraints and individual needs:

Professional 5: “When I think about intensity, I think about getting something out of the limited resources we have “… “How can we use the personnel and the time and at the same time consider patients' need of rest and also the resources they have…”… “There are a lot of patient transfers (e.g. from wheel chair to toilet) during an afternoon shift. That also has to do with intensity, all the small things we all the time consider because it is neurorehabilitation”.

Professional 2: “Intensity is a part of getting through the day and the psychological balance is another part of getting through the day”.

Therapists agreed and also gave their opinion on including more intensity both during the day and within therapy sessions:

Professional 1: “There are more layers. We would like to have increased intensity over the day, and we also want more intensity in the individual training sessions”… “Intensity is also the subjective experience – the feeling of having been immersed in a task”.

Professional 6: “...it is more obvious for the physiotherapists to regard intensity as equal to the number of repetitions”.

#### 4.1.2. After Implementing the Activity Block

All patients expressed that more training had been provided after the implementation of the Activity block:

Patient 3: “It is super good and it contributes to getting more training, because before implementing the Activity block, the two hours were just spent sitting and watching television and reading, but now there is activity and training”.

Several patients expressed the need for rest after the effort they had put in the Activity block:

Patient 4: “You are tired after lunch, and it is good to lay down a little in your room” “… just half an hour, maybe one hour, and then you are fresh again…sometimes it is enough to lay in the bed, just to get the feeling of being ready again”.

Patient 7: “I lay there for five to ten minutes - boom - then I sleep”.

Incorporating a break during or immediately after the Activity block was a consideration:

Patient3: “I think that after a training session you could have a pause where you can small talk about what you got out of it (training) and how it feels …” “It should not be optional, it should be obligatory”.

Some of the patients had aphasia and reported that this could be an additional challenge or cognitive load of their rehabilitation:

Patient 1: “We also have to use language … that is also tiresome”.

Patient 6: “You could make it (type of training) mandatory, some could train language and others could do physical training”. Several patients agreed.

All health professionals experienced a change towards more active time for the patients. One of the participants expressed it in this way:

Professional 5: “When I look at the patients' day now versus before the introduction of the Activity block, they (patients) are very much more active, in one way or another, and I think that what they receive from us, and how their day is planned, is beneficial for their rehabilitation process”.

An important reflection among the health professionals was that the patients could accommodate the extra training better than expected before implementing the Activity block:

Professional 3: “I think now that they can tolerate more than what I thought before implementing the Activity block…where before we would have included half an hour rest…”.

Professional 6: “I think generally that they have better endurance than I, than we, earlier have given them credit for”.

### 4.2. Motivation and Meaningfulness

#### 4.2.1. Before Implementing the Activity Block

Many patients highlighted the importance of motivation and factors contributing to it. In addition, the feeling of meaningfulness was emphasized. One patient expressed the importance of receiving feedback as follows:

Patient 1: “It is very important to get feedback, and that is sometimes missing”.

Being able to perform personal daily life activities independently was of outmost importance:

Patient 4: “It is our body, and I can only talk for myself, but I have no bigger wish than just to be able to independently go to the toilet again”.

Health professionals likewise emphasized motivation and ways to increase it.

Professional 5: “A big focus is to find some activities that give meaning for them”.

Professional 2: “It makes it easier to encourage the patient to put more effort in to intensity when it is perceived as meaningful for the patient”.

In order to motivate the patients, they considered the importance of using the environment as a way to make the patient be more active:

Professional 4: “The context can be influential”.

Professional 2: “Enriched environment – you can shape the environment in a way that invites to physical activity”.

Overall, there was a positive expectation towards implementation of an Activity block.

Health professionals were especially looking forward to interdisciplinary teamwork and learning from each other:

Professional 5: “I am looking forward to the interdisciplinary part of it…I think, that a lot of learning will come out of it and can be used at other times of the day”.

Professional 1: “it can be a good place for learning – to learn from each other and use it during the day” … “We will get to know the patients in another way, to see some other sides of our patients, their challenges and resources, in another context”.

#### 4.2.2. After Implementing the Activity Block

The patients expressed a positive view on being able to train and practice in a context without constantly being under the surveillance of the health professionals:

Patient 3: “It is actually good, that we now can be started up with an exercise machine or an exercise and then she (therapist) walks away and you can go on by yourself”.

Further, the patients highlighted what could motivate them:

Patient 7: “It is about to find out in each case, what is engaging instead of doing something boring all the time”.

One participant gave an example from his habitual life that perfectly illustrated this:

Patient 4: “Before I was injured, I could run further in a football game than I could on a straight road, because it has an element of game in it... “…it is the goal that makes it meaningless to quit “... it is ok for it (activity) to be boring now, but in a month's time I can walk again”.

The organization of the Activity block provided room to self-manage the intensity by deciding on when and how often to take a break during training:

Patient 3: “The Activity block is good because I can to do it in my way, on my conditions. It is of course necessary to be corrected, but I am allowed to do it in my own pace, and when I need a break, I will take a break. It is a huge degree of freedom”.

Health professionals observed that patients were taking more responsibility for their training:

Professional 3: “The patients are very motivated. They sit prepared, also before time, and I think that is because it gives meaning for them”.

Professional 5: “Some patients did actually get a little bit of ownership and said: I have to go to the toilet now, because I have to be ready for training at 10 o'clock”.

The professionals stressed the possibility to experience patients in different situations and expressed their positive view on group dynamics among themselves and the solidarity among patients:

Professional 2: “The nurses also learn a lot, because we come so close. Some tips and tricks are shared with us and that is really good. Also to see patients in another context”.

Professional 6: “I actually think it is nice to experience the community that it gives to the patients”.

### 4.3. Expectations and Concerns

#### 4.3.1. Before Implementing the Activity Block

Overall, the patient group found it important to train more during the day, and they also expressed considerations of a more individualized training:

Patient 6: “It (training) should be more differentiated. Some have a specific need for more training for example, and have a need for training in the afternoon, and the rest of us that can independently walk to the training area, can maybe train on our own”.

The health professionals also considered how to individualize the training in order for it to give meaning for the patients:

Professional 3: “You should not put them in group training just to have something on their calendar. It should be something that fits them”.

The health professionals were worried about the patients' ability to manage and take responsibility of self-training:

Professional 7: “Not everybody can initiate training by themselves. It is important that the personnel will support them to get started…and to get quality to it”.

Professional 3: “An important aspect is to keep track if they are keeping up with the self-training… “Our experience is that they are actually taking a lot of ownership in such a scheme”.

The health professionals experienced challenges in planning the day for the patients and had concerns about overall logistics and also about how patients would tolerate the Activity block:

Professional 7: “We think that we use a lot of time to coordinate the ongoing training, and it (the planning of the Activity block) is not always successful”.

Professional 5: “Not all patients can feel if they are tired in the body or if they are tired in the brain”.

#### 4.3.2. After Implementing the Activity Block

Several patients acknowledged initial challenges when starting the activity block. One said explicitly:

Patient 6: “There were some difficulties in the start…the personnel was standing and talking and discussing …the first couple of days, but it was quickly over”.

The structure of the Activity block was described in various ways. The implementation of more group training during the day was discussed in the patient group:

Patient 6: “Really, I think one block in the morning between 10-12, and another one an hour in the afternoon, and then I think you are covered, also mentally, because you also need rest”.

The health professionals highlighted structural challenges after having experienced working in Activity block for several weeks:

Professional 5: “It seems that the patients get more intensity in their training. Where they (patients) earlier have expressed that they were sitting and waiting for training until late afternoon. Now they are more active. However, providing this intensity requires some logistics”.

Professional 6: “…sometimes it is difficult to have time for the individual session, so it requires some planning and prioritizing”.

On the other hand, the health professionals experienced that some patients took a more active role in organizing their day by planning ahead of training (Activity block):

Professional 1: “Some remember that they have to go to the toilet before training and sometimes they are ready up to 20 minutes before”.

Professional 4: “…and one time they actually went over to the training area and started the training by themselves”.

Moreover, the content of the Activity block was not fixed but adapted to match the patients' overall goals:

Professional 6: “Some parts of the training are the same for everybody and some parts are actually more individual, so it differs…”.

Professional 5: “Sometimes we have started with a common warm up, where either all are making exercises on a bench or they sit in a circle and work with their hand before we spread out to the station where they are going to work”.

The health professionals described some negative aspects of implementing the Activity block:

Professional 3: “We have had some (patients) who simply enjoyed that block so much, that it has been hard to have an individual session not being a part of the Activity block”.

Nurses and occupational therapists felt that the Activity block was originally a physiotherapy domain where they had to find their role. Based on the statements from the group, it seems that they experienced a challenge to join the Activity block on the same premises as other health care professionals, i.e., physiotherapists:

Professional 3: “I still think that it sometimes is hard to come over there (Activity block) and just be there, and not just be there, but be one of them that already are engaging in the training, so I lean myself on them that seem to know more…”.

Professional 1: “It is the same for the nurses, to find our role. It is not something you just find”.

Professional 4: “There is some logistics that is supposed to be solved because we have our differences in our professions and that is good, but it requires that we work together even more with what is important and what to prioritize”.

### 4.4. Questionnaire

Patients filled a short questionnaire after the implementation of the Activity block. Results are presented below, see Table 6.

## 5. Discussion

Our aim was to explore how both patients and health professionals experience the change from mainly individual treatment to organized group treatment and to explore patients' and health professionals' expectations and experiences of participation in an Activity block.

Overall, all patients agreed that the Activity block was a good way to train, and recommended, that it should be continued. Both the participants and the health professionals experienced that the training they were offered after implementation of the Activity block had become more intense, which they appreciated. Many described increased motivation induced by the group setting. Further, self-management was found to be enhanced, and a sense of reflectiveness, e.g., to do the activity in your own pace, became obvious for both the participant groups. There were some challenges, too. Patients expressed concerns to finding a balance between rest and activity, while the health professionals mentioned practical challenges, i.e., planning the content of the day for patients, as well as finding their role as a professional in the Activity block.

Both patients and professionals expressed that training intensity could be understood in different ways. It could be interpreted as the number of repetitions trained in the same session, the number of sessions during the day, or the content of time spent together by patient and health professional. Moreover, intensity could be achieved through daily life activities, i.e., trying to speak could be intensive training for patients with aphasia. Most patients complained about too little training intensity before implementation of the Activity block, while after implementation, all participants, both patients and professionals, expressed that the patients were more active in one way or another. Too much passive time during rehabilitation has been described in numerous studies [6–8]. We did not objectively measure the amount of active time before and after the implementation, but the fact that all patients and health professionals were gathered at one location reduced waiting and transfer time would contribute to augment activity level as a result of the Activity block. Group settings are viable means of increasing activity levels as demonstrated in a study by Khan et al. [24] where all patients spend at least 2 hours a day substantially increased activity in a mixed neurological population. Also, Rosbergen et al. [25] introduced environmental enrichment including daily group sessions at an acute stroke unit with a similar promising result. It is interesting from an economical point of view that both in our and the aforementioned studies, active time could be increased within existing staff levels. Furthermore, group training does not seem to compromise safety, even if gait training is offered for rather severely impaired patients as in a study by Renner et al. [10]. Correspondingly, no adverse effects were reported in our study.

Many patients stated that participating in the Activity block provided the opportunity for self-determined training. At the same time, they emphasized the need for support from health professionals during the training. Patients were given, and they also took over, more responsibility to manage their own training. The Activity block provided time and space to explore and experience that they could manage some parts of the training on their own. This may be particularly important as many patients with stroke will have to live with long-term consequences requiring continued rehabilitation or training as secondary prevention.

Extended self-determination and decreased therapist dependency already during rehabilitation could contribute to lasting beneficial health behaviors [26]. Also, activities perceived as meaningful to everyday life are crucial and can support strengthening the construction of meaning [27]. Having time on their own to explore and feel bodily sensations, without being constantly monitored, can support the patients in perceiving that they are the ones moving their body (sense of ownership) and also being the initiator of the action (the sense of agency). Enhancement of sense of agency is considered important regarding independence in daily living [28].

Besides the amount, also the content, of training is important. Our patients stated that they need to feel challenged. The professionals expressed that they want to support the patients by targeting individual goals and challenges, also in a group setting. Exploiting experience-dependent plasticity and at the same time avoiding learned bad use should be the goal of any therapy setting, individual, group, or self-training [29]. This is even more important when training is offered during the early phase after acquired brain injury [30], as was the case for the Activity block.

Healthcare professionals both expected and experienced sharing and gaining knowledge due to their participation in the Activity block. Training with the patients and helping each other in daily situations away from the ward brought knowledge forward that the health professionals could put in to action in other times and situations, i.e., on the ward. Being together with patients and colleagues in a different setting, and on a frequent basis, resulted in an enhanced team spirit, which was conceived as valuable in other situations at the ward.

Both focus groups experienced challenges in working with the Activity block. The patients expressed more need for rest/sleep after training, but this was also perceived in a positive way. Sometimes, it was just a short rest that was necessary to get energy back and to go on with the day again. Prior to implementing the Activity block, the professionals were concerned about the amount of training that patients would tolerate during the two hours of Activity block. However, after implementing the Activity block, they experienced that the patients not only tolerated more training than expected but also took responsibility in preparing for and participating in the block.

The questionnaire sums up the experiences that the patients had being part of group training together in a joint area. Overall, they gave a positive feedback of the experiences in Activity block with regard to the contact and time with the professionals, training around other patients, the joint training area, and also the match of training with the individual needs of the patients.

## 6. Limitations and Strengths

We included all patients hospitalized in the ward at the time of data gathering which could strengthen the transferability of the findings in the current study. In addition, we strategically chose personnel from all three groups in the patients' close teams, nurses, occupational therapists, and physiotherapists, with an appropriate range in age and experience in the neurorehabilitation field. The shared positive experience of the Activity block further strengthens the transferability. Coincidentally, most of the patients were male at the time of implementation, and although we strived for heterogeneity in the professional focus group, all were female due to the composition of employees at the ward. In order to get the same patients' impressions and experiences, we gathered data from the patient group after two weeks of participation meaning that patients did not experience the Activity block for an extended period. However, we think that these two weeks were relevant to compare different training strategies. The second focus group interview of the personnel was performed after three months and is therefore based on experience over time. Type and level of aphasia and cognitive challenges were not formally assessed. Cognitive impairments could have impacted participation in the interviews and also the experience of the Activity block. This study was based on a small sample size. However, the provided information on implementation of an organized and intense training approach may inspire to adaptions in other rehabilitation settings for patients after acquired brain injury.

## 7. Conclusion

Activity block benefitted a heterogeneous group of patients with acquired brain injury and was perceived as an overall positive experience by patients and health personnel. The current study provides a deeper understanding of group training that requires complex support. Most patients and health professionals experienced positive changes regarding the intensity of training, motivation, and meaningfulness as well as in the structural framework supporting the Activity block. Some concerns linked to different aspects of the health professionals' new role, organization, and interaction with patients and colleagues were also expressed. Emphasis should be placed on matching the training to the individuals' need for support in order to find balance between rest and activity as well as using tasks that support patients' motivation.

## Figures and Tables

**Table 1:** Description of the daily overall focus in the Activity block.

Monday	Tuesday	Wednesday	Thursday	Friday
Focus on LE Walking/balance	Focus on UE, TOT; ADL-training; training of specific movement components (outdoor activities and training)	Focus on both UE and LE	Focus on LE Walking/balance	Focus on UE, TOT; ADL-training; training of specific movement components; brain education class

LE: lower extremity; UE: upper extremity, TOT: task-oriented training; ADL: activities of daily living.

**Table 2:** Interview guide May 2019.

Main categories	Questions to patients (P) and health personnel (H)
Training and intensity	P: what is your general impression of the training that you are offered now? Positive and negative aspects?
	H: what is your general impression of the training that the patients are offered now? Positive and negative aspects?
	P and H: what do think about the intensity of the training?
Training and patients' challenges and preferences	H: do you think you can match the individual's challenges?
	P: do you feel, that you are offered the type of training that matches your challenges?
	H: how do you motivate the individual patient?
	P: does the training seem meaningful to you?
Organization of training	P and H: how do you perceive progression in training?
	P and H: what are your thoughts about activity block/group training that soon is to be initiated at your ward?
	H: how are the resources distributed between patients with reduced physical and cognitive function?
	H: how do the different groups of health professionals work with the patients?

P: patients; H: health professionals.

**Table 3:** Characteristics of the patients included in interview.

Patient number	1	2	3	4	5^∗^	6	7
Sex (male/female)	Male	Male	Male	Male	Female	Male	Male
Age (years)	71	71	68	26	67	49	64
Diagnosis	Ischemic	Ischemic	Ischemic	TBI	Hemorrhage	Tumor	Ischemic
Days since injury	29	41	145	32	150	9	89

^∗^Participated only in the first interview.

**Table 4:** Characteristics of the health professionals included in interview.

Professional number	1	2	3	4	5	6
Occupation	PT	PT	OT	OT	Nurse	Nurse
Age (years)	<30	30-50	30-50	30-50	>50	>50
Neurorehabilitation experience (years)	1	22	12	15	16	5

PT: physiotherapist; OT: occupational therapist.

**Table 5:** Overview of categories.

	Training intensity
Category	Motivation and meaningfulness
	Expectations and concerns

**Table 6:** Questionnaire: “Your experience of the Activity block – group training”.

Patient number	Questions					
	I think that I am treated good by the therapists responsible for the training in the activity area	I think it is motivating to train in the same area as the other patients	I think it is disruptive to train in the same area as the other patients	I feel that the therapists responsible for the training have sufficient time for me	Joint training in activity area matches my challenges well	I think that group training in the activity area is a good training service that should continue
1.	1	2	3	1	1	1
2.	1	2	3	1	1	1
3.	1	2	2	1	2	1
4.	1	2	3	2	1	1
5.	1	1	1	2	1	1
6.	1	1	3	1	2	1
7.	2	1	1	2	1	1

1: true; 2: partly true; 3: not true; 4: do not know.

## Data Availability

No data available.
